# Effect of Field Of View on Detection of External Root Resorption in Cone-Beam Computed Tomography 

**DOI:** 10.22037/iej.2017.35

**Published:** 2017

**Authors:** Yaser Safi, Sahar Ghaedsharaf, Alireza Aziz, Sepanta Hosseinpour, Hamed Mortazavi

**Affiliations:** a *Department of Radiology, Dental School, Shahid Beheshti University of Medical Sciences, Tehran, Iran; *; b * Private Dentist, Tehran, Iran ; *; c * Research Fellow, Dental Research Center, Research Institute of Dental Sciences, Dental School, Students' Research Committee, Shahid Beheshti University of Medical Sciences, Tehran, Iran; *; d *Department of Oral Medicine, School of Dentistry, Shahid Beheshti University of Medical Sciences, Tehran, Iran*

**Keywords:** Cone-Beam Computed Tomography, External Root Resorption, Field of View

## Abstract

**Introduction:**

Conventional methods for diagnosis of external root resorption (ERR) are based on clinical findings and x-ray observations which are not appropriate for early diagnosis. The present study assessed the effect of different sizes and field of views (FOVs) in the diagnosis of simulated external root resorption by cone-beam computed tomography (CBCT).

**Methods and Materials:**

In this diagnostic *in vitro* trial, 100 human extracted mandibular central incisors were collected and marked in 3 apical, middle and coronal areas. Cavities with different sizes were created in buccal and lingual surfaces of each area. Following this procedure, CBCT images were taken in 2 different 6 × 6 cm and 12 × 8 cm FOVs with the same voxel size of 0.2 mm. Absence or presence of cavities in CBCT images were assigned by 3 radiologists and compared with gold standard results which were obtained by measurement of the size of cavities using a digital caliper. Sensitivity and specificity values, positive predictive value (PPV) and negative predictive value (NPV), A_Z_ value and Kappa values were calculated and reported.

**Results:**

Amounts of sensitivity in 6 × 6 cm FOV with voxel size of 0.2 mm for small, medium and large cavities were 95.93%, 96.03% and 97.1%, respectively. Amounts of sensitivity in 12 × 8cm FOV with the same voxel size for small, medium and large cavities were noted as 94.4%, 96.03% and 98.5%, respectively. However, specificity in FOV of 6 × 6 cm and FOV of 12 × 8 cm was calculated as 93.03% and 90.83%, respectively.

**Conclusion:**

Both used FOVs show nearly same performances in the case of detection of ERR; therefore, smaller FOV should be preferably used for detection of ERR in order to decrease the amount of imposed radiation dose given to patients.

## Introduction

External root resorption (ERR) is a multi-factorial procedure which can potentially cause irreversible loss of tooth structure and may even lead to a tooth loss [1, 2]. This complication can occur due to various reasons including periapical inflammation or lesions, traumatic occlusion, impacted teeth, traumas, tooth replantation, internal bleaching, tumor and cysts, bacterial invasion, and systemic complications or it can occur with no cause (idiopathic) [2]. Conventional method for diagnosis of this condition is based on clinical findings and x-ray observations [3]. In the primary stages, there is a chance of recovery and calcification by eliminating the irritant factor, hence an early diagnosis is very essential for an appropriate treatment [4]. Until now, conventional intraoral radiography with films or photostimulable phosphor plate, and charge-coupled device (digital radiography) are the most commonly applied radiological assessment for the diagnosis of ERR. However, conventional radiography as two dimensional imaging shows false negative results in 51.9% of cases and false positive results in 15.3% of cases [3]. As a result, previous studies revealed the lesions less than 0.3 mm in depth and 0.6 mm in diameter are not detectable by conventional periapical radiography [5, 6]. The problem arises when the lesions are located on buccal or lingual surfaces of the roots [3]. Moreover, they are able to detect the lesions after occurrence of 60-70% of demineralization [7]. Periapical radiographies are not capable of distinguishing ERR during the first months of orthodontic therapy [8]. The ability to correctly identify the location and size of the root resorption is essential for treatment planning and determination of prognosis (8). 

Therefore, three dimensional digital imaging with higher resolution can be beneficial. In endodontics, cone-beam computed tomography (CBCT) is subjected as an impressive method for assessment of endodontic complications like perforations, vertical root fractures, and dental traumas [9-12]. CBCT images are one of the most reliable tools used to study the anatomy, morphology of root canals and complications such as vertical root fractures [13], which have reduced the several limitations of two-dimensional images and which come with less imposed radiation and higher resolution in comparison with CT images [14]. The advantages of CBCT when compared with other CT scans methods are lower artifacts and real-time image analysis, and fast scanning time [15, 16]. Previous studies demonstrated the diagnostic ability of CBCT scans for ERR lesions [17-20]. However, few data are available about the factors which influence the reconstruction elements in the diagnosis of ERR. Field of view (FOV) is one of the most important determining factors in image quality [21, 22]. The aim of this study was to evaluate the diagnostic efficiency of CBCT in detection of ERR with different sizes of FOV.

## Materials and Methods

In this *in vitro *study, a total of 100 single-rooted mandibular central incisors with visible pulpal canals without restorations, endodontic therapy, anomalies and pathologies were used. All of the teeth were disinfected by 2% glutaraldehyde solution [23]. Three types of artificial external root resorptive defects were created; shallow (width: 0.6 mm, depth: 0.3 mm), moderate (width: 1.2 mm, depth: 0.6 mm), and deep (width: 1.8 mm, depth: 0.9 mm) using round burs (SS White Burs Inc., Lakewood, NJ, USA) by an experienced endodontist. A total of 75 experimental cavities (*n*=25) at three different levels (cervical, middle, and apical third) of each surface (buccal or lingual) were used for this study. The control group consists of 25 intact teeth (without artificial defect). All specimens were coated by a wax layer to decrease the artifacts [24] and mounted on an equal combination of ground rice and plaster [25]. In order to stimulate a soft tissue shadow and prevent further decrease of the artifacts, a custom made U-shaped model holding water was constructed. The allocated four groups of specimens were coded by a blind person for further evaluations. The CBCT scans were captured by NewTom VG 9000 CBCT device (Quantitative Radiology SRL Co., Verona, Italy) with 12 × 8 cm and 6 × 6 cm FOV, and 0.2 mm voxel size. Exposure parameters for both FOV sizes were 110 kVp, 5.4 sec and 0.7 mA. The images were reconstructed and evaluated in axial plane by NTT Viewer software program (NTT Software Corporation, Yokohama, Japan). Three experienced oral and maxillofacial radiologists, who were working with CBCT, blindly evaluated the presence of resorption and the qualitative grade of lesions. The answers were counted as correct only if the examiner recognized both the location and existence of the cavity.

In order to evaluate the accuracy of each FOV, MedCalc (MedCalc Software bvba, Belgium) was used to calculate the receiver operating characteristic (ROC) curves (A_z_ values) for each group. The specificity, sensitivity, negative predictive value (NPV) and positive predictive value (PPV) were calculated. The t test was used to detect statistical difference between two FOV groups in diagnosing resorption. The significance level was set at 0.05 and 95% confidence interval. All statistical analyses were performed by SPSS software (version 16.0, SPSS Inc., Chicago, IL, USA).

## Results

**Figure 1:**
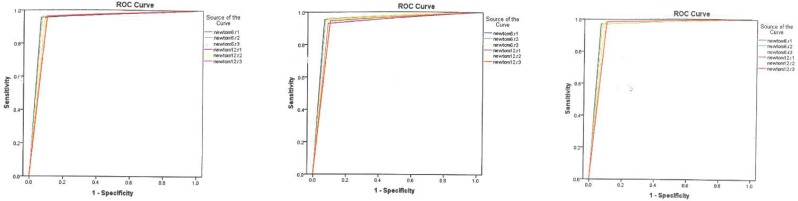
*A)* ROC curve for the first observer; *B)* ROC curve for the second observer; *C)* ROC curve for the third observer

Means of inter-class correlation coefficient for NewTom VG 9000 CBCT device was 0.992 for 6 × 6 cm FOV and 0.991 for 12 × 8 cm FOV size. In order to assess the accuracy of the test, ROC curves for each cavity size (small, medium, and large), observers (1, 2, and 3), and FOV sizes (6 × 6 cm or 12 × 8 cm) are shown in Figures 1 to 3. As the figures presented, ROC curves in all observers shown an area of 1.0. This fact indicates the test is perfectly accurate due to the high sensitivity. 

Tables 1 to 3 show overall specificity, sensitivity, and PPV for each cavity size. Overall sensitivity was defined as the detection of defect without specifying the size of defect. 

Comparison of specificity between experimental groups (FOV size: 6 × 6 cm or 12 × 8 cm) with t test showed statistically significant differences between specificity (*P*<0.05) and the two groups. However, sensitivity did not show any significant differences even in various cavity sizes (*P*>0.05) between experimental groups. It was observed that standard deviation was zero in the large cavity group, as such statistical analysis was discontinued in this group (Table 4). 

## Discussion

This study assessed the impact of CBCT images with two different sizes of FOVs in recognition of simulated ERR. As a result of non-significant differences between the two FOVs, it is better to apply CBCT evaluations with the smaller FOV size to reduce patient radiation exposure and intensify imaging contrast.

Inflammatory root resorption is not uncommon after dental trauma and can influence the survival rate of traumatized teeth. Previous studies reported the prevalence of root resorption between 5-70% of cases in different traumas like luxation, avulsion and intrusion. Moreover, replacement resorption is more common in intrusive traumas [26-28]. The early diagnosis of invasive cervical root resorption and inflammatory root resorption is a very critical step in determining the outcome of treatment and prognosis [29, 30]. Root resorptions are routinely recognized by periapical radiography with different angulations. In many cases, the nature of these images as a two dimensional scan misleads the right decision making due to inaccuracy of determining the severity, type and location of defects [31-33]. In this regard, digital radiography has presented many potential advantages in endodontic clinical practice [34]. Meanwhile, three dimensional nature of CBCT imaging can help clinicians to achieve a better diagnosis of root resorptions [35-37]. Many studies demonstrated higher accuracy of CBCT in recognition of root resorption when compared with periapical radiography [38-40]. Moreover, Lima *et al. *[41] demonstrated the statistical superiority of CBCT in root resorption diagnosis when compared with the periapical radiography in teeth after root canal therapy. However, ionizing radiation should follow the *as low as reasonably achievable* (ALARA) rule and CBCT should not be used as the first para-clinical examination for diagnosing all root resorptions. The diversity among imaging techniques, reconstruction tools, and especially exposure parameters consisting of voltage, and time of exposure can influence the detection of root resorption [42-46]. Factors like FOV, voxel, and filters used in various CBCT devices affect their diagnostic ability especially volume reconstruction tools [23, 47-50]. 

**Table 1:** Specificity, sensitivity, and PPV for small cavities

	**FOV 6 × 6 cm**			**FOV 12 × 8 cm**		
	**1st observer**	**2nd observer**	**3rd observer**	**1st observer**	**2nd observer**	**3rd observer**
**Sensitivity**	85%	90.1%	89.3%	87.8%	90.8%	90.8%
**Total Sensitivity**	95.4%	96.2%	96.2%	93.1%	95.4%	94.7%
**PPV**	84.2%	85.5%	85.4%	83.3%	84.4%	83.2%

**Table 2:** Specificity, sensitivity, and PPV for medium cavities

	**FOV 6 × 6 cm**			**FOV 12 × 8 cm**		
	**1st observer**	**2nd observer**	**3rd observer**	**1st observer**	**2nd observer**	**3rd observer**
**Sensitivity**	80.6%	83.6%	83.6%	79.2%	80.6%	80.6%
**Total Sensitivity**	95.5%	96.3%	96.3%	95.5%	96.3%	96.3%
**PPV**	87.8%	91.8%	89.6%	89.8%	91.5%	92.3%

**Table 3:** Specificity, sensitivity, and PPV for large cavities

	**FOV 6 × 6 cm**			**FOV 12 × 8 cm**		
	**1st observer**	**2nd observer**	**3rd observer**	**1st observer**	**2nd observer**	**3rd observer**
**Sensitivity**	91.2%	92%	92%	94.9%	95.6%	94.9%
**Total Sensitivity**	97.1%	97.1%	97.1%	98.5%	98.5%	98.5%
**PPV**	89.9%	91.3%	92.6%	92.2%	92.3%	91.5%

**Table 4:** Comparison of the overall specificity, sensitivity, and PPV between two FOVs group

**Variable/Group**		**Mean (SD)**	***P*** -value*
**Specificity**	**FOV 6×6 cm**	2.2 (0.43)	0.0001
	**FOV 12×8 cm**	1.8 (0.21)	
**Sensitivity for small cavities**	**FOV 6×6 cm**	1.533 (0.55)	0.1483
	**FOV 12×8 cm**	1.36 (0.21)	
**Sensitivity for medium cavities**	**FOV 6×6 cm**	1.2 (0.26)	1.00
	**FOV 12×8 cm**	1 (0.32)	

On the other hand, these differences can change the amount of absorbed radiation doses for patients [51]. To maintain the dose as low as logically needed for achieving appropriate diagnostic scans, we need to create a balance between parameters affecting the imaging quality and absorbed doses, which is still a challenging issue [43]. In the present study, the most efficient FOV size was investigated according to these facts. In accordance to our results, Silveria *et al. *[21], indicated that either small or large FOV sizes did not significantly change the accuracy of imaging. They studied both voxel size and FOV size in internal root resorption and suggested that the quality of imaging is more related to the voxel size. In addition, other studies showed similar results in different FOV sizes [52-54]. Thus, it can be recommended that for the same voxel size, smaller FOV size is more cost beneficial due to the decreasing radiation dose received by patients especially in small range like external root resorption.

Voxel resolution also can influence the diagnostic accuracy of CBCT scans in detection of internal and external root resorption [20, 55]. Liedke *et al. *[20] showed that 0.2-0.3 mm voxel resolutions significantly improve identification of resorptions when compared with 0.4 mm voxel resolution. Although 0.3 and 0.2 mm voxel resolution indicated very similar results, they recommended 0.3 mm due to the shorter scanning time it uses to reduce patients' receiving dose. In this investigation, which is an experimental *in vitro *study, we have chosen 0.2 mm. Moreover, Hatcher [56] in his study mentioned that in order to reduce noises, the lower mAs should be used and this promotes CBCT images quality. In this study, 0.7 mA was used for imaging.

## Conclusion

To evaluate external root resorption lesions, it is better to use the smallest available amount of FOV in order to reduce patient dose and enhance contrast and resolution of images. Using much smaller voxel size amounts than the depth of external root resorption cavities in comparison with using smaller voxel size amounts than the depth of external root resorption cavities, may lead to an increase in patient dose and have no influence on a more accurate diagnosis.
